# Learning to Learn Independently: Guiding Students to Develop Self-Directed Learning Skills During Medical Student Independent Research Projects—Findings from an Australian University

**DOI:** 10.1007/s40670-024-02054-4

**Published:** 2024-04-30

**Authors:** Kerry Uebel, Jane Ellen Carland, Maha Pervaz Iqbal, Greg Smith, Sally Nathan

**Affiliations:** 1 School of Population Health, Faculty of Medicine and Health, UNSW Sydney, Kensington, Australia; 2 Department of Clinical Pharmacology and Toxicology, St Vincent’s Hospital, Sydney, Darlinghurst, Australia; 3 School of Clinical Medicine, Faculty of Medicine and Health, St Vincent’s Healthcare Clinical Campus, UNSW Sydney, Darlinghurst, Australia; 4 Centre for Health Systems and Safety Research, Australian Institute of Health Innovation, Sydney, Australia; 5 Faculty of Medicine, Health and Human Sciences, Macquarie University, Sydney, Australia; 6 School of Biomedical Sciences, Faculty of Medicine and Health, UNSW Sydney, Kensington, Australia

**Keywords:** Self-directed learning, Medical student research, Mentorship, Faculty support

## Abstract

**Introduction:**

Most medical schools offer students the opportunity to conduct independent research projects in order to learn about evidence-based medicine. This study aimed to explore the experience of students, graduates, and supervisors during an independent research project through the lens of self-directed learning.

**Methods:**

Students and recent graduates were asked to complete an anonymous survey about their experiences. Semi-structured interviews were also conducted with a purposeful sample of 11 students, 14 graduates, and 25 supervisors. Interviews were recorded and transcribed. An inductive thematic analysis was conducted and themes were refined through the lens of self-directed learning.

**Results:**

Most participants agreed that the independent research project could enable students to develop valuable self-directed learning skills. Participants commented on the importance of the research mentor, faculty support structures, and membership of a research team. Participants who were not well supported described feeling distressed and isolated.

**Discussion:**

Medical student involvement in independent research projects can develop self-directed learning skills in the presence of a one-to-one mentoring relationship with a research supervisor, structured guidelines and support from the faculty, and membership of a research team. The development of self-directed learning skills should be part of the learning outcomes of any independent student research project.

**Supplementary Information:**

The online version contains supplementary material available at 10.1007/s40670-024-02054-4.

## Introduction

Many medical schools worldwide include the teaching of research skills as an essential part of their curriculum in order to promote the practice of evidence-based medicine and to support the development of clinician researchers. The Association for Medical Education in Europe (AMEE) published an extensive guideline on developing research skills in medical students [1]. This guideline outlines which research skills should be developed and makes recommendations about how they can be taught. One recommendation is for medical students to conduct an independent research project during their medical degree. This type of self-directed learning is described as important for developing key research attributes such as curiosity and critical appraisal.

Self-directed learning is an important model in medical education [2]. It is based on assumptions about the characteristics of adult learners as learners who can be actively involved in identifying their own learning needs and outcomes, selecting resources and strategies to learn, and carrying out learning plans and even assessment [2]. It is firstly, a model to be used to guide teaching and learning approaches and secondly, a strategy for empowering health professional students to become lifelong learners [3, 4]. One of the key debates in adult learning theories is whether “self-directed learning” is the correct term as it is often misunderstood as “independent learning.”

Harden and Laidlaw in their description of health professional education point out that “self-directed learning” needs to be facilitated and prefer the term “directed self-learning” [5]. They identified three important tools that are needed to facilitate independent learning: curated resources, newly developed resources, and structured study guides with content, curriculum, and activities to support students. Brydges et al. [6] prefer the term “directed self-guided learning.” These authors describe three important roles of expert facilitators in maximising learning outcomes for self-directed learning: accurately identifying students’ level of skill; providing sufficient challenge to students to facilitate the learning of new skills; and intervening when students need additional support to address a challenge.

A key challenge in medical education is to develop curricula that support students to become self-directed learners. Too little support can result in confusion and inefficient and ineffective learning [5, 7]. Students who are not supported find it difficult to choose what to learn and what resources to use, and fail to adequately monitor their own learning and areas needing further development [6, 7]. This literature strongly underlines the need for students to learn how to direct their own learning.

Although much has been published on scholarly outcomes and student satisfaction with “independent” student research projects, there has been little exploration of how these research projects contribute to developing self-directed learning. A recent realist review explored the context, mechanisms, and outcomes of scholarly projects in developing research skills among medical students, but did not examine the role of self-directed learning in mediating outcomes [8].

This study aimed to explore student and supervisor experiences and outcomes during the Independent Learning Project/Honours (ILP/Honours) programme at the University of New South Wales (UNSW) Faculty of Medicine and Health and answer the following questions:

What is the experience and outcomes for students and supervisors during their independent research year?

Can models of “directed self-learning” explain student experience and outcomes in this “independent” medical student research project?

### Context of Study

The ILP/Honours programme, at UNSW, is a mandatory research project implemented in 2006. Students conduct their own research project during a 9-month dedicated period in Year 4 of a 6-year undergraduate medical degree in the Faculty of Medicine and Health [9]. Although self-directed learning is one of eight graduate capabilities, the first 3 years of the degree are very structured. The ILP/Honours is the first opportunity for students to choose and direct their own learning for a major part of their degree. Early in Year 3, students are given information about potential supervisors and are encouraged to negotiate their own research project with supervisors of their choice. As the research project is mandatory, students who are unable to negotiate their own project by late in Year 3 are allocated, by the ILP/Honours convenor, to supervisors who have designed suitable projects. Supervisors are required to be academics or clinician researchers employed by the Faculty or affiliated with research institutes associated with the Faculty. New supervisors have access to information sessions on what to expect and how to support student researchers completing an ILP/Honours project. Students can choose to complete an ILP or, if they have suitable marks, can choose to complete Honours.

A dedicated academic ILP/Honours convenor and an education support officer together with a committee consisting of academics from various schools are in regular contact with students in both Year 3 and Year 4. They are able to support students during their negotiations with potential supervisors in Year 3 and monitor their progress in Year 4. The learning outcomes describe learning research skills but do not explicitly cover self-directed learning skills. In order to support students in learning these research skills, the committee ensures access to regular seminars on such topics as research methodology, statistical analysis, or writing a literature review, and the convenor holds regular online question and answer sessions. Students and their supervisors are also required to agree on and regularly review written milestone documents and submit these documents to the convenor. Students are given Faculty deadlines for three assessments during the year: a literature review/research proposal document early in the year, a short oral presentation to examiners and colleagues, and a report in the form of a 5000–6000-word manuscript at the end of their research project.

## Methods

We used mixed methods including a survey and semi-structured interviews to capture the experience of UNSW students, recent graduates, and current supervisors of the ILP/Honours project.

In order to document longer-term reflections on student experience, we aimed to recruit Year 6 students who had completed their ILP/Honours 2 years previously and recent graduates from the previous 5 years. Broadcast emails were sent to 1218 recent graduates from the previous 5 years who were members of the UNSW Alumni organisation and all 296 Year 6 students in 2020 inviting them to participate. They were invited to complete an online anonymous survey on their experience during the ILP/Honours year. Those who responded were then invited to participate in semi-structured interviews with the researchers (KU and MPI). To capture the experience of supervisors, we sent a broadcast email to all 206 current supervisors in April 2021 inviting them to participate in semi-structured interviews with one of the researchers (KU).

The survey questionnaire asked students and graduates to rate their satisfaction with the ILP/Honours using a Likert scale from Very Satisfied to Very Dissatisfied, and rank statements on their learnings of various research and self-directed learning skills also using a Likert scale on a scale from Strongly Agree to Strongly Disagree. The questions in this survey were based on a survey first developed in 2010 that was used to assess student satisfaction and learnings in the ILP/Honours year. Descriptive statistics were used to summarise the responses. Participants who responded to the survey were then asked if they would participate in an interview. This further response was purposefully not linked to answers to the survey to ensure confidentiality.

In the interviews with students and graduates, participants were asked questions about their experience in independent learning, skills learnt, impact on future career, and any research outcomes (see survey in Supplementary file). In the interviews with supervisors, participants were asked about their experience in supervising students and what they felt students had learnt during the project as well as research outcomes. All participants were asked to give verbal consent at the time of the interviews. Interviews were conducted by telephone, recorded, and then transcribed. An inductive thematic analysis was conducted. Initial themes were identified by one researcher and checked independently by two others on a sample of transcripts. Early themes were considered, refined, and interpreted through the conceptual lens of self-directed learning. QSR NVivo 12, a qualitative data analysis software, was used to code and manage interview data.

## Results

A total of 69 recent graduates and 52 Year 6 students completed the survey. Most of the graduates (*n* = 45, 65%) and students (*n* = 32, 62%) reported they were somewhat or very satisfied with their ILP/Honours year (Table 1). Of the graduates and students who were satisfied, 87% and 81%, respectively, reported either producing an article or conference presentation of their work compared to 17% and 45% respectively of graduates and students who were dissatisfied with their ILP/Honours (Table 1).

**Table 1:** Results from survey of recent graduates and Year 6 students of ILP/Honours project satisfaction and outcomes

	Graduates *n* = 69		Students *n* = 52	
	Very/Somewhat Satisfied *n* = 45 (65%)	Very/Somewhat Dissatisfied *n* = 24 (35%)	Very/Somewhat Satisfied *n* = 32 (62%)	Very/Somewhat Dissatisfied *n* = 20 (38%)
**Research output**				
Published or presented	39/45 (87%)	4/24 (17%)	25/32 (78%)	9/20 (45%)
**Research skills**				
Thinking scientifically	40/45 (89%)	14/24 (58%)	29/32 (91%)	8/20 (40%)
Writing a literature review	42/45 (93%)	16/24 (67%)	30/32 (94%)	16/20 (80%)
Assessing evidence in articles	40/45 (89%)	12/24 (50%)	29/32 (91%)	8/20 (40%)
Research data analysis	41/45 (91%)	11/24 (46%)	29/32 (91%)	7/20 (35%)
Writing a research report	44/45 (98%)	13/24 (54%)	31/32 (97%)	12/20 (60%)
**Self-directed learning skills**				
Time and project management	39/45 (87%)	16/24 (67%)	31/32 (97%)	10/20 (50%)
Working in a team	37/45 (82%)	10/24 (42%)	22/32 (69%)	10/20 (50%)

Graduates and students who were satisfied with their ILP/Honours were more likely to report they had learnt research skills (89–98% and 91–97%, respectively) compared with those who were dissatisfied (46–67% and 35–80%, respectively). Similarly, graduates and students who were satisfied were more likely to report they had learnt self-directed learning skills (82–87% and 69–97%, respectively) than those who were dissatisfied (42–67% and 50%, respectively) (Table 1).

### Qualitative Analysis of Interview Findings

Of the 69 recent graduates who completed the anonymous survey, 14 agreed to participate in interviews. Of the 52 Year 6 students who completed the anonymous survey, 11 agreed to participate in interviews. Twenty-five supervisors agreed to take part in interviews.

Three main themes captured the experience of interview participants with respect to self-directed learning during the ILP/Honours project. Firstly, across the three interview groupings, the ILP/Honours was seen as an opportunity to foster self-directed learning skills. Secondly, support from the supervisors, the Faculty, and the research team was critical in developing self-directed learning skills during the ILP/Honours project. Thirdly, students who did not receive this support could find their independent learning project to be a devastating experience where they struggled to learn both research skills and self-directed learning skills.

### An Independent Research Project Can Foster the Development of Self-Directed Learning Skills

Many participants commented that the ILP/Honours research project year at UNSW fostered their development of skills necessary for independent learning. The types of skills they described learning included taking responsibility for their own learning, time and project management, being resourceful, problem solving, and managing relationships within a team. One graduate described what they had learnt overall during their research year:

“…*a large aspect of what I learned was the importance of self-management and self-motivation because there isn’t someone sort of ticking your name off on a roll*” (Graduate 2).

#### Taking Responsibility by Negotiating a Project

The opportunity for students to negotiate their own project for the research year was perceived as being key to students taking responsibility and ownership of the project from the outset. Supervisors, students, and graduates alike commented on how student involvement in project development helped them understand the purpose of the work and resulted in them being motivated to undertake the work required as well as feeling a sense of achievement when the project was completed:

“*I could talk to him [the supervisor] as a peer and we could go on and sort of made the plan together…. you feel … more valuable … I don’t know, you just take ownership of this thing*.” (Graduate 8).

“…*because there’s a project that.. they take a lot of ownership of. And I think at the end of the year, they were really, really pleased with themselves and what they’ve achieved. It’s something concrete. And something of value*.” (Supervisor 15).

#### Developing Time and Project Management Skills

Many of the participants described that the research project helped them to develop skills in managing their own project and the way they used their time.

“*It’s like a really important lesson for me just to learn, like, how to use my time wisely*” (Student 3).

This was echoed in comments made by supervisors, who acknowledged that students developed project management skills as they became independent.

“*They learned some skills in terms of independence, they’ve got time management that they have to deal with…and they become quite autonomous*” (Supervisor 13).

#### Learning How to Be Resourceful and Overcome Challenges

Several participants commented on how managing problems in running their own project taught students to persevere and develop resilience and how to be resourceful in finding solutions.

“*Previously, I might have been, like, a little bit inflexible, or, you know, stress when things didn’t seem to be going the right way. But I think I’ve learnt it’s possible to have plans in place in multiple different areas to make sure that, that you get an outcome*.” (Graduate 10).

“*I think the other thing that [ILP/Honours] also taught me was how to be a bit more resourceful*.” (Student 11).

“*I think perseverance and you know having to learn how to deal with constant failures and emerging out of that unscathed*” (Supervisor 8).

#### Learning How to Communicate and Manage Relationships Within a Team

Many participants also commented on how students had learnt how to communicate and work collaboratively as a valued member in a team.

“*[I learnt] how do you ask for help when you need to and how can you contribute and help other people with their research as well*.” (Student 11).

“… *managing relationships, both with peers and colleagues and, and managing a team to try and make sure the project comes to fruition*.” (Student 10).

One of the valuable things they gained was confidence in being able to speak out and voice their opinions, even among seniors.

“…*there were a few occasions where actually, you know, ‘I really think that we should include this for these reasons’. So you know, choosing when to advocate for yourself*.” (Student 8).

#### Learning Skills That Are Valuable in the Long Term

Many students and graduates commented on the skills they had learnt that helped them after their research project with further studies and work.

“…*so it was more self-directed, which then I felt translated into phase three [Years 5 and 6 of the medical program] to help me with my, I guess, balancing, clinical and also extra-curricular and study*.” (Student 4).

Some felt more confident in their own achievements as their work was valued by the team and in the broader field of research:

“…*it was incredibly rewarding as a medical student to have done a piece of work that was seen as useful for people working in that field. …. it was a year where I got a lot of pride that I had contributed to knowledge and that gave me a lot of confidence as well*.” (Graduate 1).

The same graduate commented that they had become an ILP supervisor to students as a result of the things they had learnt during their ILP project:

“*So my experience of doing the ILP has also led me to become a supervisor for an ILP student this year so having done one myself I am very comfortable and familiar with the scope of the project and the size of the project and the ability of the students at that level*.” (Graduate 1).

### Support Needed to Promote the Development of Self-Directed Learning Skills

Students, graduates, and supervisors described three critical areas of support that were necessary to promote the development of self-directed learning skills during independent research projects: a one-on-one mentoring relationship with an experienced researcher, being embedded in a research team, and faculty support structures such as access to learning resources.

#### One-on-One Mentoring Relationship with an Experienced Research Supervisor

The importance of supervisor support to help students develop self-directed learning skills was described by many participants. Students needed supervisors to be available, to help them when problems arose and to guide them, particularly in specific tasks such as analysing their data and writing reports. Many students and graduates commented on the way supervisors provided support and the importance of this one-on-one relationship:

“*I would meet up with my supervisor almost weekly, for at least an hour or two. And those meetings were both project management meetings managing the actual project of the research study, as well as a lot of training and mentoring for me about each stage of the process*.” (Student 10).

“…*so having that one-on-one relationship was really valuable in terms of getting very specific feedback with where I was up to with my skills and how to develop those*.” (Graduate 1).

Many supervisors recognised the responsibility they had to support their ILP/Honours students. One supervisor stated that,

“…*we really owe them a duty of pastoral care to make sure that we look after them. They’re our responsibility*…” (Supervisor 1).

A number of supervisors also commented on the way they had developed their own skills. Some supervisors described learning over time how to guide students in selecting a research question that was feasible and appropriate for their level of skill and the time and resources available.

“*I think it’s kind of been a bit of a learning experience there. But I think that’s kind of part of the enjoyment of it as well, it’s kind of working out what they know. …trying to work out what that level is to pitch*…” (Supervisor 7).

A number of supervisors commented on how much they enjoyed mentoring their ILP/Honours students and watching them develop skills and own their project, while a number also mentioned how student motivation, or lack of it, was also critical:

“*We’ve had great experiences we’ve had not so great experiences in you, it all depends on the person and how committed they are*…” (Supervisor 11).

#### Research Team Support

The second critical source of support mentioned by many participants was involvement in a research team and with other students both peers and near peers: One student commented:

“*One of the main reasons why my experience was very positive, was because of the immense support I had from the non-doctor team, like from the researchers themselves, and the PhD students*.” (Student 4).

Many supervisors commented on the help they received from co-supervisors, either when they first started out as supervisors or when they had junior supervisors helping them:

“*I’ve found that choosing a good co-supervisor is important. For myself and for the students. So you know share the load at times. It can be difficult*.” (Supervisor 21).

Supervisors also commented on the importance of involving students as part the research team:

“…*it’s just an example of how we try, our research team, to integrate them into our research unit. So they’re not add-ons, they’re actually integral parts of it*.” (Supervisor 1).

#### Faculty Support

The third critical source of support identified by participants were the faculty structures in place for the ILP/Honours programme. Several key features of faculty support identified included helping students negotiate their project, providing seminars on research skills, setting up milestone reports and deadlines, training supervisors, and in providing timely interventions, particularly when student-supervisor relationships were not working.

In particular, supervisors commented on the benefit for both students and supervisors of the Faculty’s role in developing the structure of the research year, outlining expectations and providing students with access to lectures and tutorials to foster research skill development.

“*You know, that it actually is spelled out what the expectations are, and, you know, you know, when the deadlines are in advance, so, I feel like that’s quite good. And, and also having to complete the project summary at the beginning, and I think it’s actually really well run*.” (Supervisor 25).

“…*the ILP has continued to evolve. I think, personally, the evolution has been for the better. I think, the more structure, the more formal teaching around research ethics, statistics, qualitative versus quantitative statistics, you know, how to present, how to write a literature review, that actually is presented by the experts on campus, I think that’s a better thing, a better process*.” (Supervisor 6).

A student described the critical help they received from the ILP/Honours convenor who stepped in when problems arose in the relationship with their supervisor:

“*It was really good that ILP Convenor helped me a lot like otherwise I wouldn’t have survived the year or even had to repeat it….. she had a listen to the whole story*.” (Student 1).

### When Support Is Lacking Students Flounder

Some students and graduates reported that their independent learning research year became distressing and they felt isolated when they did not receive the support they needed from their supervisor.

“…*during the time when I was trying to work on the final report, I received no answers from them [supervisor or co-supervisor]. And I was terribly struggling with trying to manipulate with the data or trying to just interpret the data the whole time. Like I was so lost*.” (Student 1).

“*I was almost literally doing everything myself with very, kind of, near zero supervision, and I think there were times when I was actually struggling*.” (Graduate 9).

Equally, students and graduates who felt they received little support from the faculty reported this contributed to a lack of motivation, a poor relationship with their supervisor, and subsequently a difficult year. One graduate reported that:

“…*not really feeling like I had much … guiding hand from the faculty getting a project that would help me so ultimately I ended going into my ILP year having selected a project that I had no interest in, that I did not know anything about and had a very brief encounter with the supervisor feeling very unprepared, uncertain and unclear*.” (Graduate 11).

One supervisor voiced their concern about students being expected to conduct “independent” research.

“*But I don’t I think by making it this kind of weird, independent thing, find a supervisor, find a project, get on with it, see how you go approach.. I think that that outcome … doesn’t happen for a lot of students. A lot of students get disillusioned by the whole process and they just basically want to get to the end of it and get on with year five*.” (Supervisor 14).

## Discussion

This study explored student, graduate, and supervisor experiences of the independent research projects in the undergraduate medical degree in Australia. In addition to learning research skills, participants described the development of self-directed learning skills with supervisors, the faculty, and the research team providing the support needed for students to flourish. In our survey, a high percentage of students and graduates who were satisfied with their independent research reported they had learnt both research skills and self-directed learning skills, while in our interviews participants identified that they learnt skills such as taking responsibility for their learning, time and project management, resourcefulness, and working and communicating as a team member (Theme 1) and they valued support from their supervisor, their research team, and the faculty (Theme 2). However, students who experienced a lack of the support needed, particularly from their supervisor, floundered during their “independent” research project (Theme 3). These findings support the argument that self-directed learning is not achieved independently, rather is fostered by guidance and support.

The critical role of supervisors in developing research skills for students completing scholarly activities is well described [8, 10, 11]. This is the first study that documents their critical role in helping students develop self-directed learning skills. What is unique in the student–teacher interaction in student research projects, compared to the rest of the teaching in most medical programmes, is the one-on-one mentoring relationship necessary for students to complete their research projects (Theme 2). The support students received from their supervisors, or the lack of it, has been identified in a previous study of student satisfaction at UNSW [12]. As a result of being mentored and valued as an active colleague, students gain confidence in taking responsibility for their own projects, solving challenges, and communicating within a professional community, all of which are essential lifelong learning skills (Theme 1). Supervisors also described the teaching skills they had learnt and the rewarding experience of seeing a student learn and flourish under their guidance.

We have identified in this study that supervisors need three critical teaching skills to guide student self-directed learning during their research year: identifying the student’s level of skill; guiding them in choosing and designing a project that is doable given the student’s skills, context, and resources; and stepping in to help students when they are struggling to solve their challenges (Theme 2). These three functions of a teacher/facilitator have been described previously as important skills teachers need to learn themselves if they are to foster self-directed learning skills in students [4, 6]. Having a supervisor who is able to guide a student in negotiating their own project increases student motivation and their sense of meaningfulness and ownership of their research project and this has been shown to be another critical factor in student satisfaction [12].

For the student-supervisor relationship to work and flourish, both students and supervisors need Faculty support (Theme 2). Cornett et al. identified that one of the key mechanisms for students to learn research skills during scholarly projects was the presence of structured support from the Faculty [8]. In this study, we have also shown how important Faculty support was to help students develop their skills in self-directed learning. Access to information on potential supervisors and time and support early in Year 3 at UNSW helped students to take ownership of their learning. The completion of milestone documents and setting of deadlines throughout the year assisted students to develop time and project management skills. Access to courses provided by the Faculty also help scaffold the learning process. Supervisors had access to information sessions and learning resources their students might need. In addition, active, regular contact with a dedicated ILP/Honours convenor and support officer in the preparatory year before and during their research year gave access to personal support for students and supervisors. This was critical to ensure the Faculty could intervene if things went wrong in the student-supervisor relationship. The convenor and support officer in turn had support from members of the Year 4 committee. This type of structured support has been identified as crucial for guiding self-directed learning [5].

This work also demonstrates the importance of being a member of a research team, for both students and supervisors (Theme 2). Students valued the support of other students including their colleagues and PhD students as well as other academic professional and non-professional team members. They appreciated being seen as active valued members of the team. Supervisors appreciated the help from co-supervisors and saw the value of sharing the load of teaching students. Taylor et al. describe the importance of situated learning, emphasising that learning and thinking are social activities in a specific situation [4].

However, during independent medical student research projects, if students do not receive support from their supervisors or from the Faculty and are not in a team, they can potentially flounder and struggle to learn research skills and/or self-directed learning skills (Theme 3). This is the first study we know of that demonstrates the level of this distress in some students and this has been attributed primarily to poor support from supervisors. Equally there is some evidence in this study of the difficulty for supervisors trying to support students who appear to be poorly motivated during their research year.

The strengths of this study are that we have triangulated input from students, graduates, and supervisors, and thus been able to capture longer term impacts. This has helped document the influence of the independent research project at UNSW on development of self-directed learning skills. This is the only study that we know of that has documented the development of self-directed learning skills during independent student research projects. One limitation of the study is that the sample of participants is not representative. As participants self-selected to participate, there is likely an overrepresentation of participants who have had a very positive or very negative experience. However, this has proven beneficial as we have been able to capture a range of experiences and document both the potential and pitfalls of independent research projects. We may not have been able to capture the experience of students who were unmotivated during their research year.

## Conclusion

So-called independent medical student research projects are an important tool to develop not only research skills but also self-directed learning skills. However, it is critical to recognise that the development of these skills is facilitated by support from supervisors, the research team, and the Faculty. Support structures for both students and supervisors need to be developed, with particular focus given to the development of the student-supervisor relationship. This relationship can facilitate the development of self-directed learning skills but needs to be supported in turn by Faculty structures and by involving students in research teams. Without these supports, there is a risk that students can feel abandoned, disillusioned, and isolated. The goal of developing self-directed learners should be an explicit learning outcome of “independent” student research projects, informing students and supervisors of what students are expected to achieve in order to become lifelong learners.

### Supplementary Information

Below is the link to the electronic supplementary material.

Supplementary file1 (DOCX 62.4 KB)
