# Supplementary material for: Learning to Learn Independently: Guiding Students to Develop Self-Directed Learning Skills During Medical Student Independent Research Projects—Findings from an Australian University
Source: Med Sci Educ. 2024 Apr 30;34(4):883–90. doi: 10.1007/s40670-024-02054-4 (PMC11297219; doi:10.1007/s40670-024-02054-4)
Supplement: Supplementary file 1 — Supplementary file1 (DOCX 62.4 KB) [file 40670_2024_2054_MOESM1_ESM.docx]

**Survey of student or graduate experience, satisfaction and learning outcomes for ILP/Honours**
